# Relationship between the College Student and the Campus Club: An Evolutionary Game Theory Analysis

**DOI:** 10.3390/bs14030182

**Published:** 2024-02-25

**Authors:** Lei Duan, Zhong Wang, Guanyu Zhu, Yahui Zhang

**Affiliations:** 1 Communist Youth League Committee, Yanshan University, Qinhuangdao 066000, China; duanlei@ysu.edu.cn; 2 School of Vehicle and Energy, Yanshan University, Qinhuangdao 066000, China; wangzhong@ysu.edu.cn; 3 School of Mechanical Engineering, Yanshan University, Qinhuangdao 066000, China; zhuguanyu@stumail.ysu.edu.cn

**Keywords:** student–club relationship, evolutionary game theory, system dynamics, experiment

## Abstract

In this paper, we use an evolutionary game theory approach to build a relationship model of students and clubs for the purpose of improving student enthusiasm for participating in club activities. First, the process of the model building is introduced, which mainly includes the basic assumptions and the equilibrium point stability analysis. Based on this analysis, we find that the motivation adjustment of students and clubs is a dynamic process and that unilateral efforts alone cannot achieve an ideal result. Then, we use real data from Yanshan University to evaluate the model, the results of which indicate that the model can analyze the relationship between students and clubs effectively. Finally, we provide relevant suggestions based on the model established in this study, whereby we contribute a theoretical basis and practical guidance for how students can actively participate in clubs, as well as how clubs can better develop themselves.

## 1. Introduction

### 1.1. Background

In the continuous development of education reform, college students are increasingly participating in activities outside of the classroom. One area that has witnessed this is clubs: organizations of people with a common purpose or interest who meet regularly and take part in shared activities. Therefore, it is particularly urgent to explore the relationship between students and clubs in terms of improving the development of both sides.

From a theoretical point of view, college students—as social subjects whose psychological and physical development is not yet mature—may have excellent performance in the academic field, but they could still face the crisis of dropping out because they are not fully integrated into the social life of their institution [1]. The theory proposed by [2] suggested that individuals need to cultivate a subculture that is consistent with their respective institution through adequate social interaction in at least one sub-club to meet the need for a sense of belonging. From the perspective of empirical research, club activities are conducive to enhancing students’ sense of belonging. Students with a sense of belonging have stronger academic motivation and higher self-efficacy, and they invest more time and energy in their studies [3].

However, in real life, there are many problems with the current situation of college student club participation. Furthermore, participation that is too active or passive in club activities is not conducive to the development of college students’ comprehensive ability [4]. Some students join several clubs at the same time, and the participation and preparation of various club activities will take up a great deal of time. In addition, it is not uncommon for students to be absent from class in order to participate in club activities and deal with club affairs in class, which has a negative impact on student learning. On the other hand, students who fail to actively participate in club activities are not likely to apply what they have learned in other areas outside the classroom and to connect theory with practice [5,6].

Clubs often face the dilemma of a lack of participants despite the active organization of activities [7,8,9], which not only discourages the enthusiasm of the club organizers, but also wastes the financial investment. In addition, their interest and enthusiasm gradually fade over time; thus, they no longer participate in any further club activities or even quit the club directly, which seriously reduces and destroys the cohesion of the club and adds difficulties to the club’s management. In addition, if a club organizes activities passively, this could then lead to a large loss of students, which is also a situation that cannot meet the extracurricular needs of students [10].

Based on the analysis detailed above, there is a “conflict of interest” between students and clubs, and this “conflict of interest”—which is affected by both positive and negative states and can include academic time, ability development chance, financial investment, self-development, etc.—is dynamic.

### 1.2. Literature Review

The relationship between students and clubs has been extensively focused upon. In addition, the related pieces of the literature that have been reviewed could be classified into qualitative and quantitative studies. A healthy and sustainable relationship between students and clubs can improve the ability of students in developing themselves [11], as well as aid them in adapting to university life [12] and improving their communicative abilities [13]. Ref. [14] conducted a study in which it was finally concluded that participation in club activities has a significant positive impact on academic performance. Meanwhile, the study in [15] found that there can be a negative impact on academic achievement if students spend too much time on club activities. The conclusions of these studies, in terms of the quantitative research on the relationship between students and clubs, have mostly been based on statistical models and questionnaires. Ref. [16] collected data by means of questionnaires. The results of their research showed that high-quality club activities are more likely to stimulate the motivation of college students. Ref. [17] conducted research by drawing on Ogbu’s theory of oppositional cultures and Tinto’s theory of educational disengagement; through this approach, they obtained the conclusion that participation in clubs positively affects academic achievement. The method of multinomial logistic regression was used by [18] to analyze the impact of student motivation and ability level on the development of clubs. Regarding the impact of club activities on participants, it was found that, compared to those who did not participate, participants in club activities saw a significant increase in their literature reading, writing, and even practical skills [19,20,21].

Game theory is an advanced method that is used to analyze interactive relationships, and it has been broadly applied in many fields, including education [22,23], economics [24], management science [25], etc. We argue that there is a game relationship between students and clubs and that game theory could efficiently solve this “conflict of interest”. Certain studies have used game theory to explore the relationship between clubs and students. Ref. [26] discussed the ways in which students could be made to complete assigned club activities efficiently by employing a non-cooperative game theory approach, the results of which indicated that the rational allocation of resources can motivate students. Ref. [27] proposed using game theory to explore the problem of club formation with students. The study in [28] used a cooperative game theory approach to study the relationship between students and clubs.

### 1.3. Aim of the Present Study

The gap in the existing research lies in two aspects. First, although the student–club relationship has already received considerable attention, the present study mainly provides elaborations and analyses from statistical models or questionnaires so as to reach the conclusions. In addition, despite game theory also being utilized similarly to previous studies, few of the prior studies in the literature have explored how to achieve a dynamic equilibrium between the student–club relationship in the case of conflicts of interest and how either party can maintain their own best interests, such that students can actively participate in clubs and clubs are enabled to develop at the same time. To fill the current research gap, this paper constructs an evolutionary dynamics model based on replication dynamics. In addition, it further discusses the relationship between students and clubs using evolutionary game theory.

This paper proposes an essential framework through which to discuss the student–club relationship in the case of conflicts of interest. This is achieved by using evolutionary game theory. The contribution of this paper lies in two aspects. First, the focus of this paper is not on how individual students and clubs make decisions in a specific conflict, but on groups of students and clubs who operate in the context of repeated conflicts, wherein they constantly adjust their strategies to maximize their own interests and finally reach a dynamic equilibrium. In order to quantitatively describe this process, a student–club evolutionary dynamics model is established. This model is used to analyze the convergence direction of the student–club conflict system under different conditions, which is to say investigating whether it will converge to positive or negative outcomes. Moreover, since the assumption of evolutionary game theory is that the participants are rationally bounded, the depiction of the model will be more in line with the actual situation, and the essence of the conflicts of interest between students and clubs can be found more clearly and intuitively.

## 2. Dynamic Model of the Evolution of Student–Club Relations

### 2.1. Basic Assumptions

The form of the game theory approach applied is contained in the following elements: the participating groups set, the students, and the clubs. The strategy spaces of the students are AP and PP, where AP indicates active participation and PP indicates passive participation. At the same time, the strategy spaces of clubs are AO and PO, where AO indicates active organization and PO indicates passive organization. Both of these sets of parameters obtain the corresponding reward when choosing the respective strategy.

Under normal circumstances—where the students are not in active participation or passive participation and the clubs are not in active organization or passive organization—the students and clubs will obtain the underlying rewards *P* and *V*, which always exist, regardless of the active or passive strategy chosen. The combinations of different strategies chosen by the students and clubs only result in different losses relative to the underlying reward. When the students choose PP and clubs choose PO, the students cannot improve and clubs will not be as attractive to students, which represents the situation where the students and clubs may have incurred a development loss. These are represented by *J* and *K*, respectively. When the students choose PP and clubs choose AO, which represents the situation where students negatively participate while the clubs organize positively, the enthusiasm of the clubs is damaged, thus reducing the chances of students participating in club activities in the future. The students in this situation will incur a chance loss, which is represented by *M*. When the students choose AP and clubs choose PO, which represents the situation where the students positively participate while the clubs organize negatively, the clubs see a reduction in their exposure chance among the students compared with those clubs that actively organize activities. As such, the clubs will incur a chance loss, which is represented by *N*. When the students choose AP and clubs choose AO, which represents the situation where the students may invest considerable time (i.e., an occupation of their academic time) and clubs may invest considerable funds, then the students and clubs will incur an investment loss; this situation is represented by *R* and *S*.

The gain and loss variables can be obtained, as shown in Table 1. Based on the above assumptions, a game matrix for both players under different strategies, which is achieved via deducting the loss based on the underlying rewards *P* and *V* under different choices, is presented in Table 2.

We let *x* (0≤x≤1) represent the probability of the students choosing AP, and we let (1−x) denote the probability of choosing PP. In addition, we denote *y* (0≤y≤1) and (1−y) as the probabilities of the clubs choosing AO and PO, respectively. When the probability is 1, this means that the students must be in active participation and clubs must be in active organization. When the probability is 0, this means that the students must be in passive participation and that the clubs must be in passive organization. The explanation of the probability can be understood as indicated in Table 3.

### 2.2. Modeling

According to the above assumptions, the expected gain of the students was divided into two parts: one part was the gain $U_{11}$, where the students chose AP, and the other was the gain $U_{12}$, where the students chose PP. Finally, the total gain was denoted by $U_{1}$. The equations for the above are as follows:

(1) $$U_{11}=\left(P-R\right)y+P\left(1-y\right)=P-Ry,$$

(2) $$\begin{array}{cc} U_{12} & =(P-M)y+(P-J)(1-y)\\ \; & =P-J+(J-M)y, \end{array}$$

(3) $$\begin{array}{c} U_{1}=xU_{11}+\left(1-x\right)U_{12}\\ \begin{array}{cc} & =x(P-Ry)+(1-x)(P-J+(J-M)y) \end{array}\\ =P-J-(J+R-M)xy+Jx+(J-M)y, \end{array}$$

The critical point of evolutionary game theory is in the dynamic changes in strategy probabilities. The replication dynamics equation effectively explains this relationship between the different game agents [29]. The basic form of the replication dynamics equation in evolutionary game theory is $\frac{\partial Z}{\partial t}=p\left(U_{\exp}-U_{\mathrm{aexp}}\right)$, where $\frac{\partial Z}{\partial t}$ represents the rate of change in the game subject’s strategy choice over time, *p* is the probability of making choices, $U_{\exp}$ is the expected utility of the relevant strategy, and $U_{\mathrm{aexp}}$ is the average expected utility.

According to (1)–(3) and the above definition, the replication dynamics equation for the students choosing AP is as follows:

(4) $$\begin{array}{cc} F_{1}\left(x,y\right) & =\frac{\partial x}{\partial t}=x\left(U_{11}-U_{1}\right) \end{array}$$

The expected gain of clubs can be divided into the gain in choosing AO with $U_{21}$ and also into the gain of choosing PO with $U_{22}$. Moreover, the total gain is denoted by $U_{2}$. The equations for the above are as follows:

(5) $$U_{21}=\left(V-S\right)x+V\left(1-x\right)=V-Sx,$$

(6) $$\begin{array}{cc} U_{22} & =(V-N)x+(V-K)(1-x)\\ \; & =V-K+(K-N)x, \end{array}$$

(7) $$\begin{array}{c} U_{2}=yU_{21}+\left(1-y\right)U_{22}\\ \begin{array}{cc} & =y(V-Sx)+(1-y)(V-K+(K-N)x) \end{array}\\ =V-K-(K+S-N)xy+Ky+(K-N)x, \end{array}$$

According to (5)–(7) and the above definition, the replication dynamics equation for the clubs choosing AO is as follows:

(8) $$\begin{array}{cc} F_{2}\left(x,y\right) & =\frac{\partial y}{\partial t}=y\left(U_{21}-U_{2}\right)\\ \; & =y\left(1-y\right)\left(U_{21}-U_{22}\right)\\ \; & =y(1-y)(K-(K+S-N)x), \end{array}$$

## 3. Evolutionary Equilibrium Stability Analysis

### 3.1. Equilibrium Point Calculation

In order to find the equilibrium points of the student–club game system, all of the equilibrium points can be found according to the concept of the evolutionary stability strategy and the knowledge of differential equations:

(9) $$\left\{\begin{array}{c} \partial x/\partial t=0\\ \partial y/\partial t=0 \end{array}\right.\;x,y\in \left[0,1\right],$$

Regardless of the value of the payment variables *M*, *R*, *N*, and *S*, there are four sets of definite solutions for the second-order differential equations of (0,0), (0,1), (1,0), and (1,1). When *M < R* and *N < S*, there is another set for a possible solution (*K*/(*K* + *S* − *N*),*J*/(*J* + *R* − *M*)).

The solution of the above situation is the equilibrium points of the student–club game system, and whether they are stable points or not needs to also be judged.

The Jacobian matrix of the student–club game system is

(10) $$J=\left(\begin{array}{c} \frac{\partial F_{1}\left(x,y\right)}{\partial x},\frac{\partial F_{1}\left(x,y\right)}{\partial y}\\ \frac{\partial F_{2}\left(x,y\right)}{\partial x},\frac{\partial F_{2}\left(x,y\right)}{\partial y} \end{array}\right).$$

In order to analyze the stability of the equilibrium points, the possible equilibrium points were substituted into the Jacobian matrix, and the determinant and trace of the Jacobian matrix that corresponded to the different equilibrium points were calculated, as shown in Table 4.

### 3.2. Equilibrium Points Stability Analysis

If the determinant of the matrix is greater than 0 and the trace of the matrix is less than 0, then the system is in a steady state at that equilibrium point. At the same time, the values of the determinant and the trace of the matrix are related to the loss variables of each participating individual in the two groups. For the same equilibrium point, the value of the loss variable was different, the values of the determinant and trace of the Jacobian matrix were different, and the type of equilibrium point was also different. Therefore, considering the different values of the loss variables, we can judge the types of the equilibrium points in various cases, and we can also analyze the process and results of the evolutionary game, as shown in Table 5.

The factors that determine the determinant and the trace of the matrix are, in the main matrix, the size of the variables *M* and *R*, as well as *N* and *S* in the game matrix. Considering that the sizes of *M* and *R* are mainly *M < R* or *M > R* and that the sizes of *N* and *S* are mainly *N < S* or *N > S*, then we can combine them into four cases to discuss the types of equilibrium points in the different cases.

First of all, we briefly discuss the practical significance of the four situations in which *M* and *R*, as well as *N* and *S*, are combined.

From the perspective of the clubs, if a club actively organizes activities when the number of students involved is small, then it will result in a waste of resources to a certain extent. Conversely, if there is lack of activities being organized by a club, then it will reduce the unnecessary loss to a certain extent and the investment loss would be greater than the chance loss, thus indicating *N < S*.

From the perspective of the clubs, if a club actively organize activities when the number of students involved is large, then it will meet the needs of students to a certain extent. Conversely, if there is a lack of activities being organized by a club, then it will result in student dissatisfaction with the club and, to a certain extent, it will increase unnecessary loss, which means that the investment loss would be less than the chance loss, thus indicating *N > S*.

From the perspective of the students, if students are actively participating in activities when there are fewer activities being organized by a club, then it will decrease student enthusiasm and interest to a certain extent due to the more passive organization. If there is a lack of students participating in activities, then it will reduce unnecessary loss to a certain extent and the investment loss would be greater than the chance loss, thus indicating *M < R*.

From the perspective of students, if students are actively participating when there are more activities being organized by a club, then they can choose more activities, which will stimulate student interest to a certain extent. Conversely, if there is a lack of students participating in activities, then their own development would be mitigated to a certain extent and the investment loss would be less than the chance loss, thus indicating *M > R*.

The following discussion focuses on the stability of equilibrium points in the context of four different categorization criteria.

#### 3.2.1. *M < R* and *N < S*

As shown in Table 6, the chance loss for both students and clubs was found to be lower than their respective investment losses.

As shown in Table 6, it can be seen that the chance loss was found to be less than the investment loss for both the students and clubs, which led to the emergence of five equilibrium points within the system. There existed two ESS points—(0,1) and (1,0)—and two unstable points—(1,1) and (0,0)—in the system, with an equilibrium point—(*K*/(*K* + *S* − *N*),*J*/(*J* + *R* − *M*))—serving as the center point. In other words, when the students passively participate in club activities and the clubs passively organize club activities, then the system cannot have a stable point. This aspect is not discussed further below.

A set of values with *J* = 1, *R* = 6, *M* = 4, *K* = 2, *S* = 8, and *N* = 4 that satisfied the current conditions (*M < R* and *N < S*) were utilized. The evolutionary path of the student–club game system was plotted under different initial (*x*,*y*) values, where *x* and *y* were selected from the vector [0, 0.1, 0.2, 0.3, 0.4, 0.5, 0.6, 0.7, 0.8, 0.9, 1]. The evolutionary path was then analyzed accordingly.

**Figure 1:**
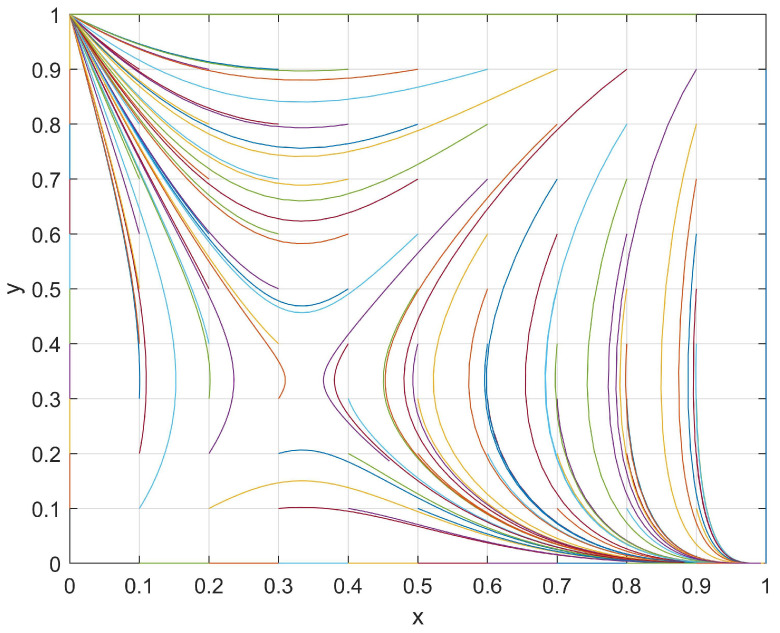
The evolutionary path of *x* and *y* when *M < R* and *N < S*.

By taking the evolutionary path with the initial values of (0.6, 0.9) as a representative value through which to analyze Figure 1, the evolutionary direction was clearly identified. At this point, both the students and the clubs exhibited relatively high levels of enthusiasm for participation in club activities. With respect to the students, when a club actively organizes club activities and if the students find that the “investment loss, *R*” is greater than the “chance loss, *M*” (*M < R*), then they will be more inclined to passively participate in club activities that reduce their loss in this situation, thus leading to a decrease in their engagement in club activities. With respect to clubs, when students actively participate in club activities, then a club may find that the “investment loss, *S*” is greater than the “chance loss, *N*” (*N < S*). In this situation, a club would be more inclined to passively organize club activities so as to reduce their loss, thus leading to a decrease in their engagement in organizing activities. At position (*x*, *y*) = (0.35, 0.85), the enthusiasm of students with respect to participating in club activities was found to be low, but the enthusiasm of clubs in terms of organizing club activities remained relatively elevated.

With respect to students, when a club exhibits a high level of enthusiasm in organizing club activities and if the students continue increasing their engagement, then they will discover that the “investment loss, *R*” is greater than the “chance loss, *M*” (*M < R*). Therefore, students tend to decrease their engagement in club activities so as to reduce loss. Regarding clubs, the engagement of students in club activities would already be low in this situation. If a club further decreases its enthusiasm for organizing club activities, then it would incur a “development loss, *K*”. Therefore, such a club would be inclined to increase its enthusiasm in organizing club activities so as to avoid development loss. The final evolutionary process converged toward (0,1), thus indicating that, regardless of how actively the clubs organized club activities, the students did not take part.

#### 3.2.2. *M < R* and *N > S*

When the chance loss is smaller than the investment loss for students and the chance loss is greater than the investment loss for clubs, then the system has the following four equilibrium points: (0,0), (0,1), (1,0), and (1,1). The stability of each equilibrium point is shown in Table 7.

**Figure 2:**
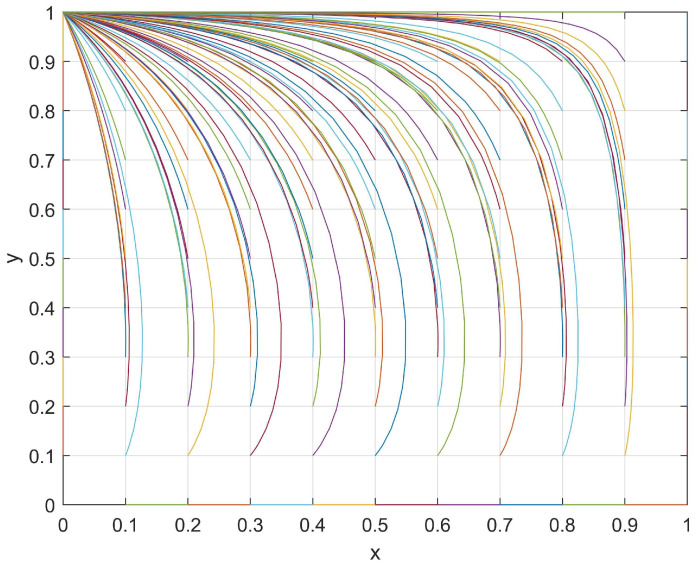
The evolutionary path of *x* and *y* when *M < R* and *N > S*.

A set of values with *J* = 1, *R* = 6, *M* = 4, *K* = 2, *S* = 4, and *N* = 8 that satisfied the current conditions (*M < R* and *N > S*) were used and the evolutionary path was shown in Figure 2. The evolutionary path of the student–club game system in these current conditions was plotted under different initial (*x*,*y*) values, and its evolutionary patterns will be discussed in the following section.

With respect to the students, when a club passively organizes activities, then the students may find that reducing their levels of participation in such activities will result in an additional “development loss, *J*”. Therefore, students tend to increase their level of participation in order to avoid this additional loss. However, when a club actively organizes activities, then students may find that the “investment loss, *R*” is more important than the “chance loss, *M*” (*M < R*). In this case, students are more likely to reduce their level of participation in order to minimize their loss. As such, the enthusiasm of students with respect to participating in club activities will decrease.

Regarding clubs, when students passively participate in club activities, a club may find that reducing their level of enthusiasm in organizing such activities will result in an additional “development loss, *K*”. However, when students actively participate in club activities, then a club may find that the “investment loss, *S*” is smaller than the “chance loss, *N*” (*N > S*). Therefore, regardless of the enthusiasm of students with respect to participating in club activities, a club would be more inclined to reduce its loss through actively organizing club activities. As a result, the level of enthusiasm in organizing club activities by a club will also increase.

In conclusion, the system exhibits the ESS point (0,1) during its evolutionary process, whereby the students passively participate in club activities while a club actively organizes activities.

#### 3.2.3. *M > R* and *N < S*

When the chance loss is greater than the investment loss for students and the chance loss is smaller than the investment loss for clubs, the system has the following four equilibrium points: (0,0), (0,1), (1,0), and (1,1). The stability of each equilibrium point is shown in Table 8.

**Figure 3:**
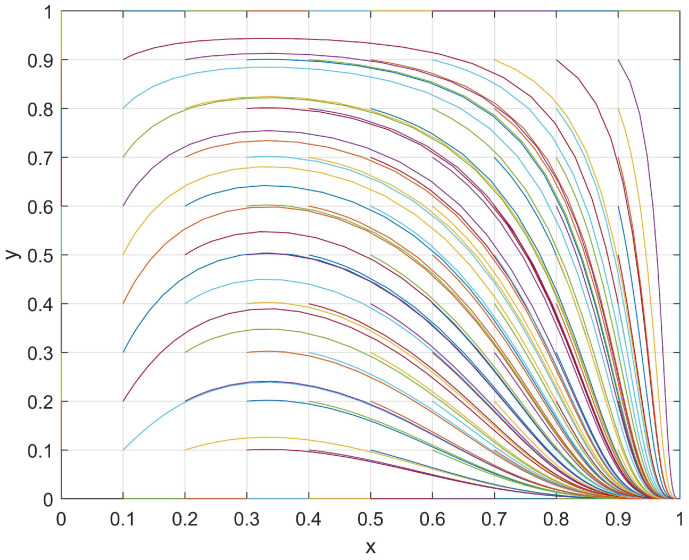
The evolutionary path of *x* and *y* when *M > R* and *N < S*.

At this point, one stable point (1,0) and two saddle points (0,1), (1,1) were observed in the student–club game system. Subsequently, a set of values with *J* = 1, *R* = 4, *M* = 6, *K* = 2, *S* = 8, and *N* = 4 that satisfied the current conditions (*M > R* and *N < S*) were used and the evolutionary path was shown in Figure 3. The evolutionary path of the system in these current conditions was plotted under different initial (*x*,*y*) values, and its evolutionary patterns are discussed in the following section.

Regarding students, when a club passively organizes activities, the students may find that reducing their levels of participation in such activities will result in an additional “development loss, *J*”. However, when a club actively organizes club activities, then students may find that the “investment loss, *R*” is smaller than the “chance loss, *M*” (*M > R*). Therefore, regardless of the level of enthusiasm shown by a club in organizing club activities, students will be more inclined to take an active part in order to minimize their loss. As a result, the levels of participation and enthusiasm for students in club activities will increase.

With respect to clubs, when students passively participate in club activities, a club may find that reducing their level of enthusiasm in organizing such activities will result in an additional “development loss, *K*”. Therefore, clubs may still prefer enhancing their enthusiasm in organizing club activities so as to reduce loss. However, when students actively participate in club activities, a club may find that the “investment loss, *S*” is greater than the “chance loss, *N*” (*N < S*). In this situation, a club would be more inclined to passively organize club activities in order to reduce its loss, thus leading to a decrease in its engagement in organizing activities.

In conclusion, the system exhibited the ESS point (1,0) during its evolutionary process, which is when students actively participate in club activities while a club passively organizes activities.

#### 3.2.4. *M > R* and *N > S*

When the chance loss for both students and clubs is greater than their respective investment loss, the system has the following four equilibrium points: (0,0), (0,1), (1,0), and (1,1). The stability of each equilibrium point is shown in Table 9.

**Figure 4:**
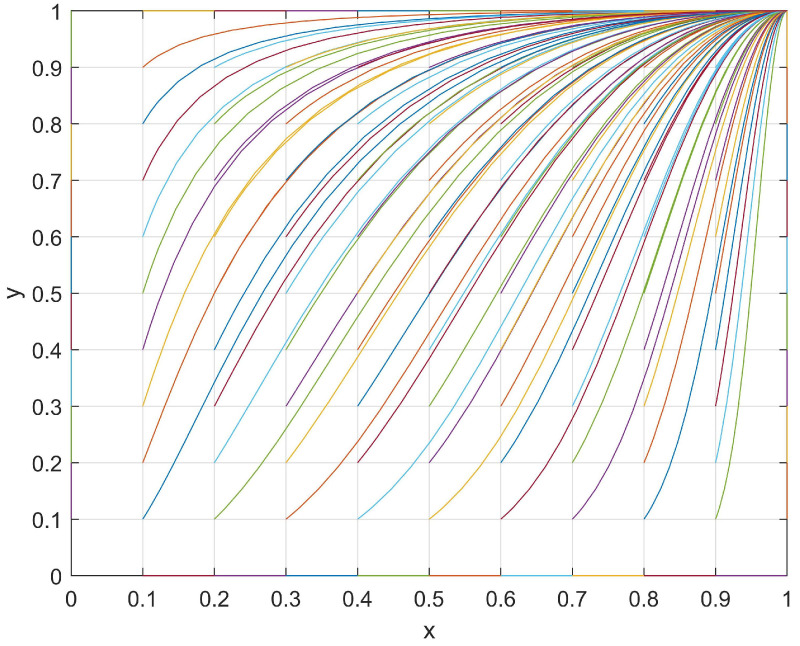
The evolutionary path of *x* and *y* when *M > R* and *N > S*.

At this point, one stable point (1,1) and two saddle points (0,1), (1,1) were observed in the student–club game system. Subsequently, a set of values with *J* = 1, *R* = 4, *M* = 6, *K* = 2, *S* = 4, and *N* = 8 that satisfied the current conditions (*M > R* and *N > S*) were used and the evolutionary path was shown in Figure 4. The evolutionary path of the student–club game system in the current conditions was plotted under different initial (*x*,*y*) values, and its evolutionary patterns are discussed in the following section.

With respect to students, when a club passively organizes activities, the students may find that reducing their levels of participation in such activities will result in an additional “development loss, *J*”. However, when a club actively organizes club activities, students find that the “investment loss, *R*” is smaller than the “chance loss, *M*” (*M > R*). Therefore, regardless of the level of enthusiasm shown by a club in organizing club activities, students are more inclined to actively participate in order to minimize their loss. As a result, the levels of participation and enthusiasm for students in club activities will increase.

Regarding clubs, when students passively participate in club activities, a club may find that reducing its level of enthusiasm in organizing such activities will result in an additional “development loss, *K*”. However, when students actively participate in club activities, a club may find that the “investment loss, *S*” is smaller than the “chance loss, *N*” (*N > S*). Therefore, regardless of the enthusiasm of students in participating in club activities, a club would be more inclined to reduce its loss through actively organizing club activities. As a result, the level of enthusiasm in organizing club activities by the clubs will also increase.

In conclusion, the system exhibited the ESS point (1,1) during its evolutionary process, which is when students actively participate in club activities and clubs actively organize activities.

### 3.3. Experiment Analysis

We collected data on the club organizations and student participation of Yanshan University from 2018 to 2022. The following verify the effectiveness of the model through actual statistical data. In fact, only the relative size relationships between *M* and *R* as well as *N* and *S* need to be quantified, and the resulting trend is the same as the convergence trend in the model. However, in practice, it is difficult to match the parameters; as such, we quantified the relative size of the parameters via questionnaires.

We researched a total of nine club-related types of information and numbered each club, using C1, C2, C3,..., C9, etc., to represent the names of the clubs. We quantified the student enthusiasm through the number of students participating in the clubs. At the same time, we quantified the enthusiasm of the clubs through the funds invested. Moreover, we further verified the effectiveness of the model through the relevant information of these nine clubs. For the sake of illustration and verification, we grouped C1–C3, C4–C6, and C7–C9 together when the convergence trend of these groups was the same as that of the theoretical model, and this group was represented separately during the simulation.

**Figure 5:**
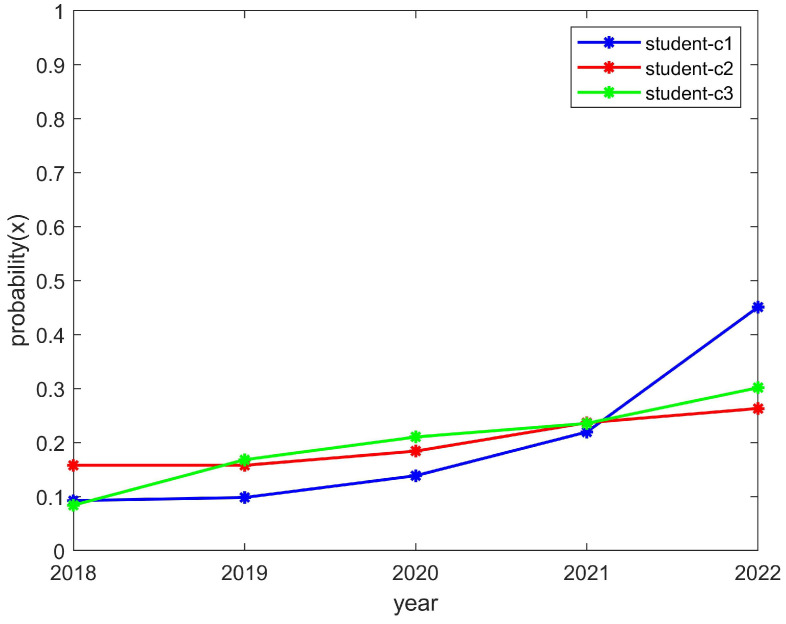
The enthusiasm trend of students who participated in the C1, C2, and C3 clubs.

**Figure 6:**
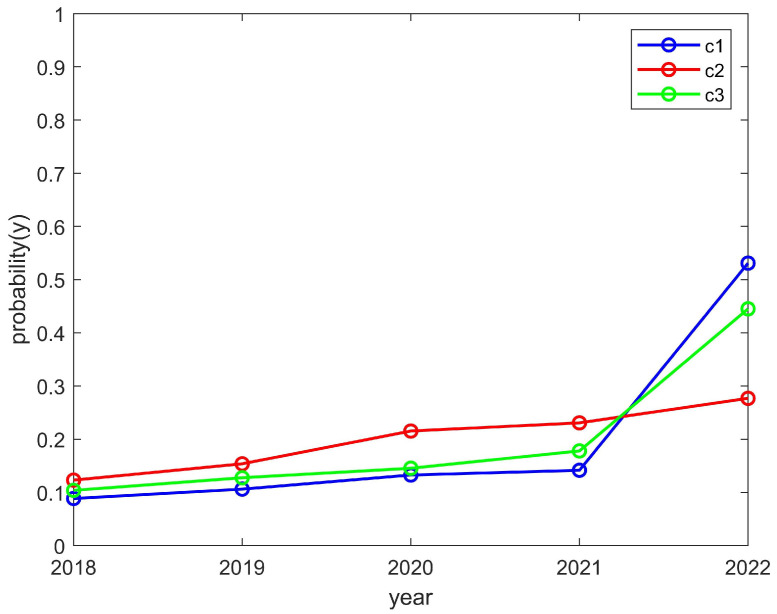
The enthusiasm trend of the C1, C2, and C3 clubs.

As shown in Figure 5 and Figure 6, the enthusiasm of students and clubs showed an upward trend, which is consistent with the trend of the above model converging to (1,1). The reason for this was that the chance loss of students and clubs is greater than the investment loss. In other words, students can reasonably control their spare time and their development will not be affected by participating in clubs, which helps with promoting the enthusiasm for the organizing of activities by clubs. While its exposure is guaranteed, a club should also have sufficient funds to hold activities; thus, this should promote student enthusiasm for participation in clubs. As a result, student and club motivation would be on the rise.

**Figure 7:**
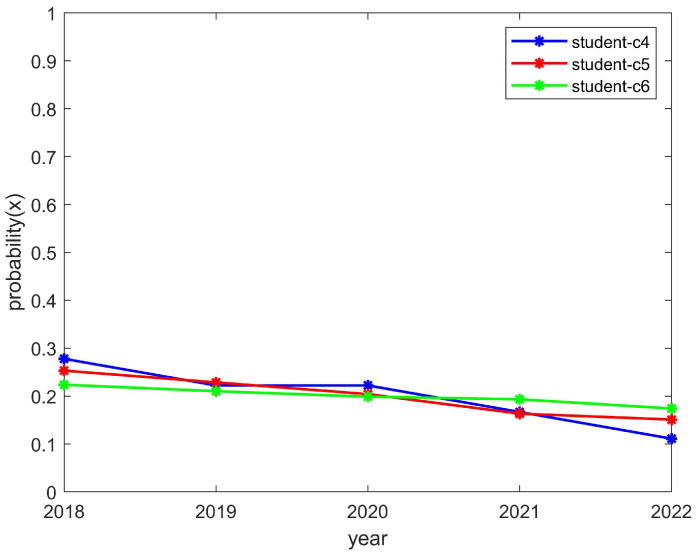
The enthusiasm trend of students who participated in the C4, C5, and C6 clubs.

**Figure 8:**
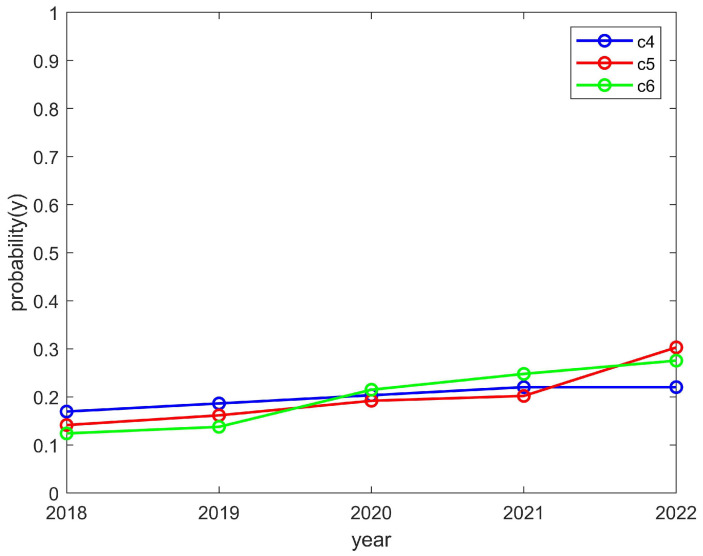
The enthusiasm trend of the C4, C5, and C6 clubs.

The students’ enthusiasm showed a downward trend and the clubs’ enthusiasm showed an upward trend, as shown in Figure 7 and Figure 8, respectively. This is consistent with the trend of the above model converging to (0,1). The reason for this is that, if students find they are wasting their time by participating in club activities, then their enthusiasm will decrease. However, if a club reduces its organizational enthusiasm, then it will hinder its own development; thus, such a club would likely increase its organizational enthusiasm. If this is the case, the enthusiasm of students will decrease and the enthusiasm of clubs will increase.

**Figure 9:**
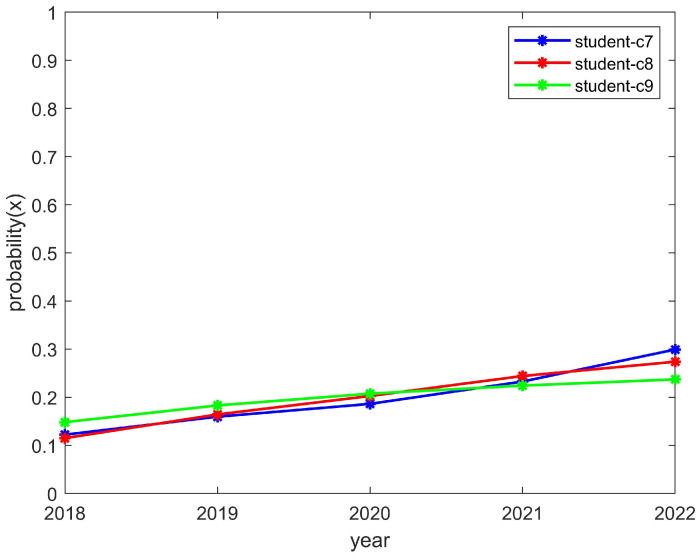
The enthusiasm trend of students who participated in the C7, C8, and C9 clubs.

**Figure 10:**
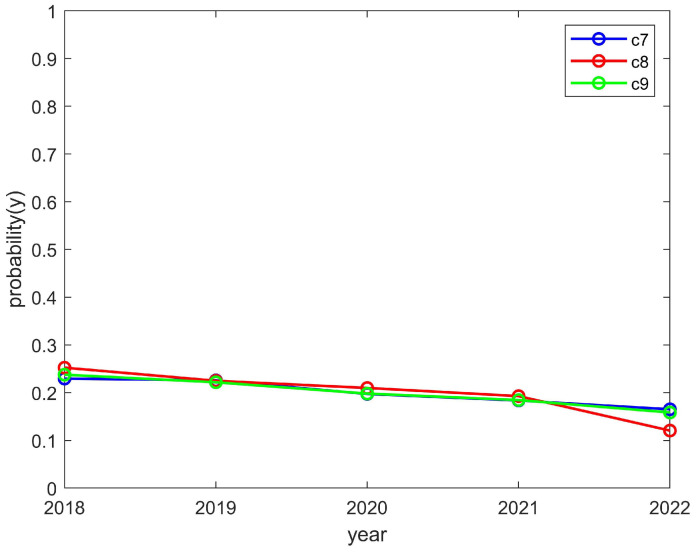
The enthusiasm trend of the C7, C8, and C9 clubs.

The students’ enthusiasm showed an upward trend and the clubs’ enthusiasm showed a downward trend, as shown in Figure 9 and Figure 10, respectively. This is consistent with the trend of the above model, which converged to (1,0). The reason for this was that students can reasonably allocate their time to participate in activities. However, a club may have to reduce the number of activities it organizes due to funding issues and, thus, would be in a state of passive organization. In such a case, students would still actively participate in order to encourage the club to organize more activities later and thus improve the enthusiasm for the club to organize more activities in the future. As a result, the student motivation would increase while the club motivation would decrease.

### 3.4. Discussion

In summary, the evolutionary process between the students and clubs was determined by the investment loss, chance loss, and development loss. Figure 1, Figure 2, Figure 3 and Figure 4 show that the motivation adjustment of the students and clubs is a dynamic process and that the motivation trend of students and clubs will be different under different variable conditions. At the same time, the enthusiasm of the students and clubs only converged to (1,1) under the conditions of *M > R* and *N > S*, which indicates that unilateral efforts alone cannot achieve an ideal result. Only when students believe that participating in a club can bring better development to themselves and when clubs believe that their input costs can be rewarded will both of them have the opportunity to form a good promotion and circulation relationship. Based on the above analysis, we make the following suggestions:

1. Improve the organization process of club activities:
  Club activities should be based on the interests and needs of students themselves, thus allowing students to participate in club activities according to their individual requirements;
2. Cultivating distinctive cultural activities in clubs:
  A campus should strictly screen club cultural activities and shift their focus toward organizing high-quality cultural activities in order to establish a positive cultural image;
3. Increasing the promotion efforts of club cultural activities:
  Clubs should innovate their promotional methods, continue to strengthen the promotion efforts of activities, and enhance the enthusiasm of students for participating in campus cultural activities;
4. Establishing a feedback mechanism for club cultural activities:
  To ensure the progress of cultural activity, clubs should establish a reasonable feedback mechanism. This can be achieved by collecting feedback information and the objective evaluation of students, among other such methods. By doing so, this will reflect the real situation of club cultural activity progress and continuously improve the content based on student demands.

## 4. Conclusions

In this paper, we explored the relationship between students and clubs based on an evolutionary game theory approach. In addition, we clarified that investment loss, chance loss, and development loss are the key influencing factors that determine the evolution of students and clubs. The analysis showed that the motivation adjustment of students and clubs is a dynamic process and that unilateral efforts alone cannot achieve an ideal result. At the same time, we counted the relevant data for theoretical verification. The following four aspects were detailed based on the above analysis: the organization process of club activities, the cultural activities in clubs, the promotion efforts of club cultural activities, and the feedback mechanism for club cultural activities. These four aspects provide a theoretical basis and practical guidance for how students can actively participate in clubs and how clubs can better develop themselves.

## 5. Limitations and Future Research

In this study, we argue that the focus of this paper is not on how individual students and clubs make decisions during a specific conflict, but rather on clubs and groups of students that operate despite repeated conflicts. In future work, we will develop a model using a single club and multiple students who make different decisions to support our work.

## Figures and Tables

**Table 1:** The gain and loss variables.

Variable Name	Student	Club
Underlying Reward	*P*	*V*
Development Loss	*J*	*K*
Chance Loss	*M*	*N*
Investment Loss	*R*	*S*

**Table 2:** Game matrix.

Strategy Combination	Student Benefit	Club Benefit
(AP,AO)	*P − R*	*V − S*
(AP,PO)	*P*	*V − N*
(PP,AO)	*P − M*	*V*
(PP,PO)	*P − J*	*V − K*

**Table 3:** Probabilities of the possible scenarios.

*x*	*y*	Realistic Meaning
1	0	Students must be in AP and clubs must be in PO
0	1	Students must be in PP and clubs must be in AO
1	1	Students must be in AP and clubs must be in AO
0	0	Students must be in PP and clubs must be in PO

**Table 4:** The determinant and trace of the Jacobian matrix that corresponded to the different equilibrium points.

Equilibrium Points	Determinant of the J	Trace of the J
(0,0)	*JK*	*J + K*
(0,1)	*K(R − M)*	*M − R − K*
(1,0)	*J(S − N)*	*N − S − J*
(1,1)	*(R − M)(S − N)*	*R − M + S − N*
(*K*/(*K* + *S* − *N*),*J*/(*J* + *R* − *M*))	z*	0

Note: z* = [*−JK(S − N)(R − M)*]/[*(K + S − N)(J + R − M)*].

**Table 5:** The discriminant of the equilibrium points type.

Equilibrium Points	Determinant of the J	Trace of the J	Points Type
$E_{1}$	−	Uncertain	SP *
$E_{2}$	+	+	UP *
$E_{3}$	+	−	ESS *
$E_{4}$	+	0	CP *
$E_{5}$	0	+	UP *
$E_{6}$	0	Non-positive	SP *

* Note: SP, saddle point; UP, unstable point; CP, center point; and ESS, evolutionary stable strategy.

**Table 6:** The discriminant of equilibrium points type when *M < R* and *N < S*.

Equilibrium Points	Determinant of the J	Trace of the J	Points Type
(0,0)	+	+	UP *
(0,1)	+	−	ESS *
(1,0)	+	−	ESS *
(1,1)	+	+	UP *
(*K*/(*K* + *S* − *N*),*J*/(*J* + *R* − *M*))	+	0	CP *

* Note: UP, unstable point; CP, center point; and ESS, evolutionary stable strategy.

**Table 7:** The discriminant of the equilibrium points type when *M < R* and *N > S*.

Equilibrium Points	Determinant of the J	Trace of the J	Points Type
(0,0)	+	+	UP *
(0,1)	+	−	ESS *
(1,0)	−	Uncertain	SP *
(1,1)	−	Uncertain	SP *

* Note: UP, unstable point; SP, saddle point; and ESS, evolutionary stable strategy.

**Table 8:** The discriminant of the equilibrium points type when *M > R* and *N < S*.

Equilibrium Points	Determinant of the J	Trace of the J	Points Type
(0,0)	+	+	UP *
(0,1)	−	Uncertain	SP *
(1,0)	+	−	ESS *
(1,1)	−	Uncertain	SP *

* Note: UP, unstable point; SP, saddle point; and ESS, evolutionary stable strategy, ESS.

**Table 9:** The discriminant of the equilibrium points type when *M > R* and *N > S*.

Equilibrium Points	Determinant of the J	Trace of the J	Points Type
(0,0)	+	+	UP *
(0,1)	−	Uncertain	SP *
(1,0)	−	Uncertain	SP *
(1,1)	+	−	ESS *

* Note: UP, unstable point; SP, saddle point; and ESS, evolutionary stable strategy.

## Data Availability

The data are available from the corresponding authors upon reasonable request.
